# Predictors in ASD: The Importance of Parents' Perception

**DOI:** 10.3389/fpsyt.2021.506148

**Published:** 2021-08-26

**Authors:** Alda Mira Coelho, Virgínia da Conceição

**Affiliations:** ^1^Child and Adolescent Psychiatry, Faculty of Medicine, University of Porto, Porto, Portugal; ^2^Department of Psychology, Institute of Public Health, University of Porto, Porto, Portugal

**Keywords:** parent's concerns, predictors, autism spectrum disorder, early intervention, autism

## Abstract

Several predictors may influence children's developmental trajectories with Autism Spectrum Disorder (ASD), and parents' concerns may play an important role. This study aimed to investigate developmental trajectories of two groups of children with ASD to understand predictive factors, including parental perception. We examined the clinical features of a sample of 55 children with ASD at 3 and 6 years of age in two moments of evaluation to understand this process. We used the Autism Diagnostic Observation Schedule, (ADOS) in both moments. We selected two groups based on ADOS results at moment two: one group with a worse outcome (ADOS results above 8) and one group with a better outcome (ADOS results below 8 in the second moment). We also selected questions from a questionnaire (elaborated by the authors and used in clinical practice) applied to parents to understand if early parents' concerns may help to predict ASD prognosis. We found a significant association between imitation and playability and the child's prognostic. Also, Interactive Gestures, Beginning of Joint Attention, Reciprocity, and Pleasure in Interaction might help identify positive case evolution. Our findings are significant in early intervention program development, not only with direct intervention with the child but also including the parents' involvement in the intervention.

## Introduction

The Diagnostic and Statistical Manual of Mental Disorders (DSM 5) describes autism as a developmental disorder characterized by severe and pervasive impairment in several areas of development, including reciprocal social interactive skills, communication skills, and stereotyped behavior, interests, and activities.

ASD (Autism Spectrum Disorders) includes Autistic disorder, Asperger Syndrome, and Pervasive Developmental Disorders not otherwise specified (PDD-NOS). Still, they are not individualized in DSM-5, which provides for only ASD in different levels of severity. It is difficult to predict the disease evolution in early childhood. A marked impairment in emotional competence and social interaction is noted from early stages because emotions are essential to regulate social interactions, which, in turn, influence emotional development. A substantial body of research suggests that children with ASD express emotions differently, and there are autism-specific deficits in emotion perception and understanding (1). There are no consistent indications of a correlation between emotional competencies, social competencies, and ASD subtypes (2). It would be interesting to study some of these features with reliable instruments to define prognosis in the early stages (3).

During the second and third years of life, symptoms of autism usually spread to multiple areas of functioning. While typical infants develop a remarkable growth in social, communication, and imaginative play competence, infants with autism show syndrome-specific difficulties in these areas (4, 5). Developmental theory links imitation and play, and these two areas may represent a core impairment in ASD (6). They can help discriminate children with ASD from other disabilities from a very early age (7). We believe that these areas may be improved with parents' training to stimulate interaction and social communication, and we think parent's concerns may help in early diagnosis and intervention.

Charman (8) demonstrated that early joint attention and imitation, measured at 20 months of age, were related to social and communication evaluated with Autism Diagnostic Interview-Revised (ADI-R) at 42 months. It has also been found that initial IQ and language at age six were associated with the Adaptive Behavior composite score of the Vineland Adaptive Behavior Scale (VABS) (9) at age 14. Charman (8) enhanced the many significant associations between Non-verbal IQ language and ADI-R, reciprocal social interaction and Non-verbal Communication scores at age 3, and communication and socialization scores of VABS at age 7.

Very early intervention may help to improve existing difficulties and prevent or attenuate subsequent neurodevelopmental disturbances arising from early impoverished socio-emotional interactions in the first years of life (10). Unfortunately, early diagnosis and specific strategies related to early intervention in ASD are still tricky. Understanding the nature and timing of symptoms may be critical in predicting developmental trajectories within ASD and contributing to early diagnosis and intervention planning (11).

This study examined the clinical features of 55 children with ASD, aiming to understand some aspects that can predict different developmental trajectories in children with ASD, including parent's perception. Children were evaluated at 3 and 6 years of age with ADOS and parents' questionnaires.

The current study investigates developmental trajectories of two child groups with ASD trying to understand some predictive factors in order o adjust better intervention strategies.

## Methods

### Participants and Procedure

We included a total of 55 children with ASD in this study. These children were evaluated at three years of age and again evaluated at 6 years of age. The children were recruited from a Psychiatric child consultation in a General Hospital in Porto, Portugal.

The study is part of an investigation work approved by the Centro Hospitalar de São João's Health Ethics Committee. The study complies with the relevant national and institutional committees' ethical standards on human experimentation and the Helsinki Declaration of 1975, as revised in 2008. All parents signed an informed consent according to the Helsinki and Oviedo Conventions.

Diagnosis of ASD was initially given by independent clinicians (psychiatrists, pediatricians, and psychologists) with many years of experience.

DSM-5 was used for diagnosis at the time sampling around 2 years of age.

All children were evaluated with ADOS as a symptom of severity measure. We also gave parents a questionnaire (semi-structured interview elaborated by the authors and used in clinical practice) to understand their first concerns related to their children to understand if these concerns may predict clinical evolution. All children were evaluated 3 years later with ADOS. We selected two groups based on the global results of ADOS at moment two: in the best group were included all children with scores below 8, and in the worst group were included all children with scores above 8. These cut-offs are in line with the classification algorithm for autism and autism spectrum.

All the children had a psychoeducational intervention, at least 4 hours per week, with educator, occupational, and speech therapy, and they had no associated co-morbidity. It was not the purpose of this study to evaluate the type of intervention, and the use and type of medication were not considered in this study.

We selected the variables that were more relevant according to clinical features and literature (4, 5, 7, 8): Joint (or Shared) Attention, Reciprocity, Interactive Gestures, and Pleasure in Interaction (in ADOS). We also investigated potential correlations of ADOS results with the parent's questionnaire.

### Instruments

The Autism Diagnostic Observation Schedule (ADOS) (12) is an instrument for diagnosing and assessing autism. The protocol consists of several structured and semi-structured tasks that involve social interaction with the examiner to assign the subject's behavior and relate it with predetermined observational categories and quantitative scores associated with ASD.

All the evaluations were conducted by psychologists with an ADOS specialization (from the University of Barcelona), strictly following the author's instructions.

The parents' questionnaire consisted of a semi-structured interview, developed explicitly for these study, based on clinical features, with 30 questions (open and closed) to understand the parent's perspective about the moment of concern (age), type of concern, supports involved, and child's evolution. We only used closed questions (yes or no) about specific symptoms like Social Impairment, Communication (verbal and non-verbal), and Imitation and Playability to identify some potential prognosis predictors.

### Data Analysis

Statistical analyses were performed using SPSS software®. Descriptive analysis was performed using proportions in categorical variables and means and standard deviations in the continuous variables with normal distribution. The Fisher Exact test was used to test the significance of the associations, the Pearson and Spearman tests were used for the correlations between the variables, and we also used Logistic Regressions. We considered 0.05 as the level of significance.

## Results

### Participants

A sample of 55 participants was analyzed in this study, with 46 male and 9 female children. The 55 children with ASD were evaluated at 3 years of age and again at 6. Participants' average age at the time of the first evaluation was 3.4 (SD = 0.55) years for girls and 3.8 (SD = 0.44) years old for boys. At the second evaluation, the mean age of the participants was 6.5 (SD = 1.46) and 7.3 (SD = 1.73) years old for girls and boys, respectively.

### ADOS

In the two time periods analyzed, we observed a significant decrease in the mean ADOS score of the second evaluation (*p* < 0.001) (Table 1).

**Table 1:** ADOS results obtained in both evaluation moments.

	**Mean (SD)**	***p***
**Total ADOS 1**	11.29 (3.588)	<0.001
**Total ADOS 2**	8.58 (2.14)	

*Significant results at the confidence level of 95% confidence*.

In the ADOS evaluation, the relationship between the score in the first evaluation and the total score in the second evaluation was marked in the interview process carried out with the parents: Beginning of Joint Attention and Pleasure in Interaction.

Regarding the item of Shared Attention, it was verified that it does not significantly impact the total score of the first evaluation (χ^2^ = 0.15, *p* = 0.13), with results divided in the middle, as we can see in Table 2.

**Table 2:** Score frequency per item in children with ADOS total >8.

	**Evaluation 1**		**Evaluation 2**	
	**0**	**>0**	**0**	**>0**
**Joint attention**	51% (*n* = 27)	49% (*n* = 26)	19% (*n* = 5)	81% (*n* = 21)
**Pleasure in interaction**	44% (*n* = 23)	56% (*n* = 30)	8% (*n* = 2)	92% (*n* = 24)

However, this association becomes significant over time, presenting an essential indicator of a good prognosis for the second evaluation. Only 19% of the children with a null score in this item in the first evaluation scored over 8 in the second evaluation (χ^2^ = 17.46, *p* < 0.001).

Regarding the Pleasure in Interaction item, we observed a similar phenomenon, with no statistically significant association between the result of the item in the total score of the first evaluation, but with the association gaining robustness over time, with a χ^2^ = 23.21, *p* < 0.001, in relation to the total of the second evaluation, where only 8% of the results equal to 0 in this item at the time of the first evaluation had a total score higher than 8 at the time of the second evaluation.

Both items correlate significantly with the ADOS total in the second moment of evaluation, representing the item Pleasure in Interaction as a more robust predictor, r > 0.7 (Table 3).

**Table 3:** Correlation values between the first evaluation results in the identified items and the total of the second evaluation.

	**Total ADOS 2**	**Joint attention**	**Pleasure in interaction**
**Total ADOS 2**	1	0.68^**^	0.77^**^
**Joint attention**		1	0.74^**^
**Pleasure in interaction**			1

*Significant results at the confidence level of 95% confidence*;

** *p < 0.001*.

We did not find a statistically significant association between the items Interactive Gestures and Reciprocity in the total of the second ADOS evaluation. Still, we can observe significant correlations between items, as shown in Table 4.

**Table 4:** Pearson's correlation values between the first evaluation results and the total of the second evaluation.

	**Interactive gestures**	**Joint attention**	**Reciprocity**	**Pleasure in interaction**	**ADOS 2**
**Interactive gestures**	1	0.59^**^	0.73^**^	0.65^**^	0.69^**^
**Joint attention**		1	0.71^**^	0.74^**^	0.68^**^
**Reciprocity**			1	0.73^**^	0.74^**^
**Pleasure in interaction**				1	0.77^**^

*Significant results at the confidence level of 95% confidence*;

** *p < 0.001*.

### Parents' Questionnaire

It was found that the parents who identified earlier changes correspond to the cases with worse evolution before the child's one year of age. In the milder cases, parents cared more about the difficulty in speaking or socializing from the age of 2 years. There was essential concern with language and socialization (the answer yes means there is inadequate behavior). The results agree with the data identified in clinical practice and the ADOS. It was verified that the milder cases did not show apparent limitations at 2 years either in imitation ability or pleasure in playing with the adult.

In the questionnaire applied to the parents, it is possible to verify two indicators with a significant association with ADOS evaluation in the second moment: Imitation and Play, as shown in Table 5. Thus, parents' perceptions of the child's behavior seem to be better with lower instrument scores.

**Table 5:** Frequency of results in percentage, by a total of ADOS in the second moment of evaluation.

		**ADOS**		
		**>8**	**<8**	**X^**2**^**
**Imitation**	**Adequate**	56.0%	93.1%	***p*** **<** **0.05**
	**Inadequate**	44.0%	6.9%	
**Playability**	**Adequate**	36.0%	82.8%	***p*** **<** **0.05**
	**Inadequate**	64.0%	17.2%	
**Social impairment**	**Adequate**	96.6%	96.0%	*p* = 0.71
	**Inadequate**	3.4%	4.0%	
**Communication**	**Adequate**	38.7%	41.4	*p* = 0.53
	**Inadequate**	61.3%	58.6%	

*Significant results with a 95% confidence level are shown in bold*.

In the logistic regression, Playability and Imitation were included as predictors of a good result on ADOS evaluation, and the results were significant for this regression: χ2_(2)_ =10.91, *p* < 0.01. Thus, the Omnibus test presents statistically significant results, thus confirming the predictive value of these variables in ADOS' second evaluation. The Hosmer and Lemeshow test showed the value of X^2^ = 4.67 and *p* = 0.67, demonstrating the importance of the Imitation and Playability variables as predictors.

## Discussion

We noticed that the best outcome group had better scores on all items in the first and second evaluation, which may have a predictive value. In the worse outcome group, we always found severe impairment in socialization and communication. Still, we also found impairment in Playability and Imitation in moment 1, which results are different from the best outcome group. High stability has been reported for clinical diagnosis made by expert professionals, supported by standard criteria for ASD, as reported in other studies (8).

These findings are similar to other studies. Charman (2005) (8) demonstrated many significant associations between language and ADI-R Reciprocal Social Interaction and Non-verbal Communication scores, at age 3, and VABS Communication and Socialization domain scores at age 7.

The results from our study suggest that some scores observed with ADOS evaluation: Interactive Gestures, Beginning of Joint Attention, Reciprocity, and Pleasure in Interaction, might help with identifying developmental trajectories, namely, of a favorable prognosis, for children with lower scores in the specified items, independently of the total obtained in the scale.

In this study, parents' Imitation and Playability perceptions of the child are also robust possible predictors of favorable developments, as we can see in Table 5. Few studies have evaluated the relationship of early parental concerns with prognosis. Ozonoff et al.'s (6) study highlights the importance of early parental concerns' with certain early behaviors that may have predictive value in diagnosing ASD. Sacrey (13), in 2015, draws identical conclusions through a study in which a questionnaire was applied to parents of children at risk of ASD. All of them were evaluated at three years to identify the highest and lowest risk.

These data point to the need to evaluate such items considering the information given by the parents and the observation in consultation.

It also draws attention to the need and advantage of screening tests at pediatric and family doctor visits to detect early warning signs and provide timely guidance to at-risk children and their parents to begin early intervention.

Landa (12) enhanced that evidence suggests that young children with ASD benefit from early intervention and programs to teach parents to implement child-interaction strategies with parent-coaching supervising.

According to Vivanti (14), the response to early intervention in autism is variable. It reflects the need to report data about the association between predictor and outcome variables because we know so little about this. We need reliable instruments to measure what is related to adaptive capacities and educational strategies and evaluate previous family structures and resources to understand the difference in the outcome.

Cohen (9) studied a retrospective analysis of the trajectories of adaptive skills and severity symptoms in ASD subgroups and confirmed the prediction that each subgroup had different trajectories depending on the type of adaptive behavior. His finding suggests that this instrument may have a helpful prognostic value for clinical application.

Based on these results, coupled with the data from the literature highlighting the importance of the early development of social competencies (1, 7, 8), we can conclude that early diagnosis and intervention are essential. However, integrating the parents' contribution throughout the process is also very important. From our clinical experience, we observe that when the parents are involved from the beginning in therapy, with acceptance and adequate understanding of the diagnosis, the whole intervention runs better and more successfully.

These findings may be helpful to implement parent training interventions that help parents interact and communicate with their toddlers with ASD, participating throughout all the processes of daily life. We can also understand these findings as a good alternative to promote the development of their child's social and communicative skills (12) and could de be an essential contribution to their clinical evolution.

Further investigation is necessary to replicate results and develop more reliable instruments to define subtypes and prognosis early.

One limitation of this study is the sample size that might be considered small and with a heterogeneous group of children, meeting different profiles. Nevertheless, our study had rigorous diagnostic criteria and only included diagnosed ASD children, confirmed by clinical observation. It could be helpful in further research, with a larger sample, to add another instrument in the second evaluation to compare the results to correlate with possible predictive elements. Also, this study did not evaluate the effect of the intervention on the outcome of groups. It could be interesting in future studies to analyse controlled variables related to family resources and type of intervention. As it was not the purpose of our research and due to the small sample size, we did not detail and analyse the type of pharmacological treatment of the patients. Evaluations represent a particular moment in time, which needs to be considered when interpreting and generalizing results. Further investigation is necessary to replicate results and to develop more reliable instruments to define subtypes and prognosis in the early stages.

## Data Availability Statement

The datasets presented in this article are not readily available because they are confidential confidential but they can be made available upon request to the parents with mediation through the authors. Requests to access the datasets should be directed to: alda.coelho@chsj.min-saude.pt.

## Author Contributions

All authors contributed to the article and approved the submitted version.

## Conflict of Interest

The authors declare that the research was conducted in the absence of any commercial or financial relationships that could be construed as a potential conflict of interest.

## Publisher's Note

All claims expressed in this article are solely those of the authors and do not necessarily represent those of their affiliated organizations, or those of the publisher, the editors and the reviewers. Any product that may be evaluated in this article, or claim that may be made by its manufacturer, is not guaranteed or endorsed by the publisher.
